# Potential Tool Use by Wolves (*Canis lupus*): Crab Trap Pulling in Haíɫzaqv Nation Territory

**DOI:** 10.1002/ece3.72348

**Published:** 2025-11-17

**Authors:** Kyle A. Artelle, Paul C. Paquet

**Affiliations:** ^1^ Center for Native Peoples and the Environment, Department of Environmental Biology State University of New York College of Environmental Science and Forestry Syracuse New York USA; ^2^ Heiltsuk Integrated Resource Management Department Bella Bella BC Canada; ^3^ Department of Geography University of Victoria Victoria BC Canada

**Keywords:** animal behavior, canid cognition, *Canis lupus*, tool use

## Abstract

The cognitive abilities of canids are increasingly recognized, though insights from noncaptive populations are comparatively rare. Recently, recurring damage to crab traps used by Haíɫzaqv Nation Guardians to control a European Green Crab invasion was investigated with remote cameras. A wolf was recorded emerging from the water carrying a crab trap buoy, then sequentially pulling the attached line up the beach until an initially submerged trap emerged from the water. The wolf then extracted the bait cup from within and consumed the bait. The recorded behavior, combined with similarly extracted and damaged traps nearby, suggests a sophisticated understanding of the trap and sophisticated cognition more broadly. This observation raises questions about the context and origins of the behavior and prompts consideration of our relationship with this cognitively complex species.

## Graphical abstract

Recently, recurring damage to crab traps used by Haíɫzaqv Nation Guardians to control a European Green Crab invasion was investigated with remote cameras. A wolf was recorded emerging from the water carrying a crab trap buoy, then sequentially pulling the attached line up the beach until the initially submerged trap emerged from the water, with the wolf then removing the bait from within. The capacity for this sophisticated behavior reveals insights into canid cognition while raising questions about how the preconditions for such behaviors arise in this and other populations and species.

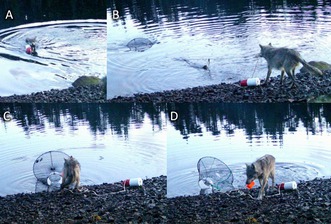

Tool use in nonhuman animals has garnered considerable scholarly attention (e.g., Emery and Clayton 2009; Bentley‐Condit and Smith 2010; Shumaker et al. 2024). Once regarded as a uniquely human attribute signifying a qualitatively superior and distinct form of intelligence, tool use is now recognized as prevalent across a diverse range of taxa (Bentley‐Condit and Smith 2010), with the complexity of tool use varying among species. Tool use is consistent with and an extension of optimal foraging: associated with increased food provisioning in species exhibiting such behavior (e.g., sea otters ( *Enhydra lutris* ; Law et al. 2024), New Caledonian Crows (*Corvus moneduloides*; Rutz et al. 2010), and capuchin monkeys (*Cebus* sp.; Izar et al. 2022)), but not necessary in other species due to different adaptations (Hansell and Ruxton 2008). Alternative foraging strategies might require cognition comparable to or surpassing that required of tool use, suggesting that tool use alone may not be a reliable indicator of animal intelligence (Shumaker et al. 2024). Moreover, many animals that do not habitually use tools, and may be presumed to lack the capacity for tool use, demonstrate tool use under circumstances where it is advantageous (Emery and Clayton 2009). Accordingly, caution should be exercised in interpreting the absence of habitual tool use as a direct indication of their capacity to use tools or of inferior cognitive ability (Emery and Clayton 2009; Shumaker et al. 2024). These cautions notwithstanding, novel observations of tool use or similarly sophisticated behavior in nonhuman species might warrant consideration, as they may provide valuable insights into potentially unexplored dimensions of those species' adaptive behavioral capabilities.

Tool use has disproportionately been observed in domestic animals (Haslam 2013), presumably because (a) these animals' subsidized and/or secure lives permit more time for exploratory behavior, and/or (b) they are observed far more often by humans, meaning even occasional tool use would be considerably more likely to be observed. Therefore, tool use when observed in nondomesticated environments might be particularly noteworthy.

The intelligence of canids is increasingly well recognized. Research across taxa further substantiates the intellectual sophistication that people have long perceived in familiar dogs ( *Canis familiaris* ). For example, several documented behaviors suggest dimensions of abilities comparable to chimpanzees ( *Pan troglodytes* ), including learning human words, and following human gestures (Smith et al. 2012). Despite behavioral complexity exhibited in other dimensions, canids have historically not been considered tool users (Smith et al. 2012). However, recent observations of captive wild dingoes ( *Canis lupus dingo* ) moving objects to then stand upon and attain objects out of reach or gain better views have been described as tool use (Smith et al. 2012). Accounts of apparent tool use in domestic dogs have also been described, including carrying hockey pucks with plastic flying discs (Shumaker et al. 2024), shaping bones into back scratchers (Bekoff 2018), and moving chairs to access food (Bekoff 2014). However, we are not aware of reported tool use by canids outside of captivity.

Beginning in 2023, crab traps used in a Haíɫzaqv Nation‐led program to control a European Green Crab ( *Carcinus maenas* ) invasion (White (Q̓íx̌itasu) et al. 2024; https://coastalfirstnations.ca/resources/managing‐invasive‐green‐crab‐in‐hai%C9%ABzaqv‐territory/) were repeatedly damaged in an area near Bella Bella, Haíɫzaqv Territory, in present‐day British Columbia. The exact location of these traps remains confidential in accordance with data sharing agreements with the Haíɫzaqv Nation and to safeguard these wolves. These traps comprise rigid frames enclosed by netting, with plastic baited cups affixed within. They have been continuously employed since 2021, initially using only herring as bait, with an additional bait type, Steller sea lion ( *Eumetopias jubatus* ) carcass portions, introduced in 2023. The extent of damage varied, ranging from minor netting tears to complete trap destruction, with all exhibiting at least some damage to the bait cups. Damaged traps were mostly deployed in the intertidal zone, exposed during low tides and submerged during high tides. Although bears (*Ursus* sp.) or gray wolves ( *Canis lupus* ) appeared to be potential perpetrators, some damaged traps were in deeper water, submerged at all tides, leading to speculation that the damage might have instead been caused by marine mammals such as pinnipeds ( *Phoca vitulina* or *Eumetopias jubatus* ) or otters (*Enhydra lutris or Lontra canadensis*).

To determine the species responsible, as part of ongoing efforts to prevent such incidents, a pilot set of remote cameras was aimed at traps where damage had occurred, deployed initially from May 28 to May 30 2024. Almost immediately (May 29), a wolf was recorded, at a mid‐to‐high tide, emerging from the water carrying a buoy attached to a crab trap line in her mouth. In rapid succession, she carried the buoy up the beach, dropped it, descended the beach, picked up the line, and pulled it farther up the beach until a trap partly emerged from the water. She then dropped the line, descended the beach again, picked up the line, and pulled the trap farther up the beach. Subsequently, she picked up the trap with her mouth and carried it to shallower water. Through the trap's netting, she chewed on and manipulated the bait cup until it fell from its attached lid. In the following recorded sequence, the bottom netting of the trap had been torn open and the bait cup removed, carried in her mouth. She dropped the cup, consumed the bait within, and then departed. The encounter lasted 3 min (Figure 1, Video 1).

**FIGURE 1:**
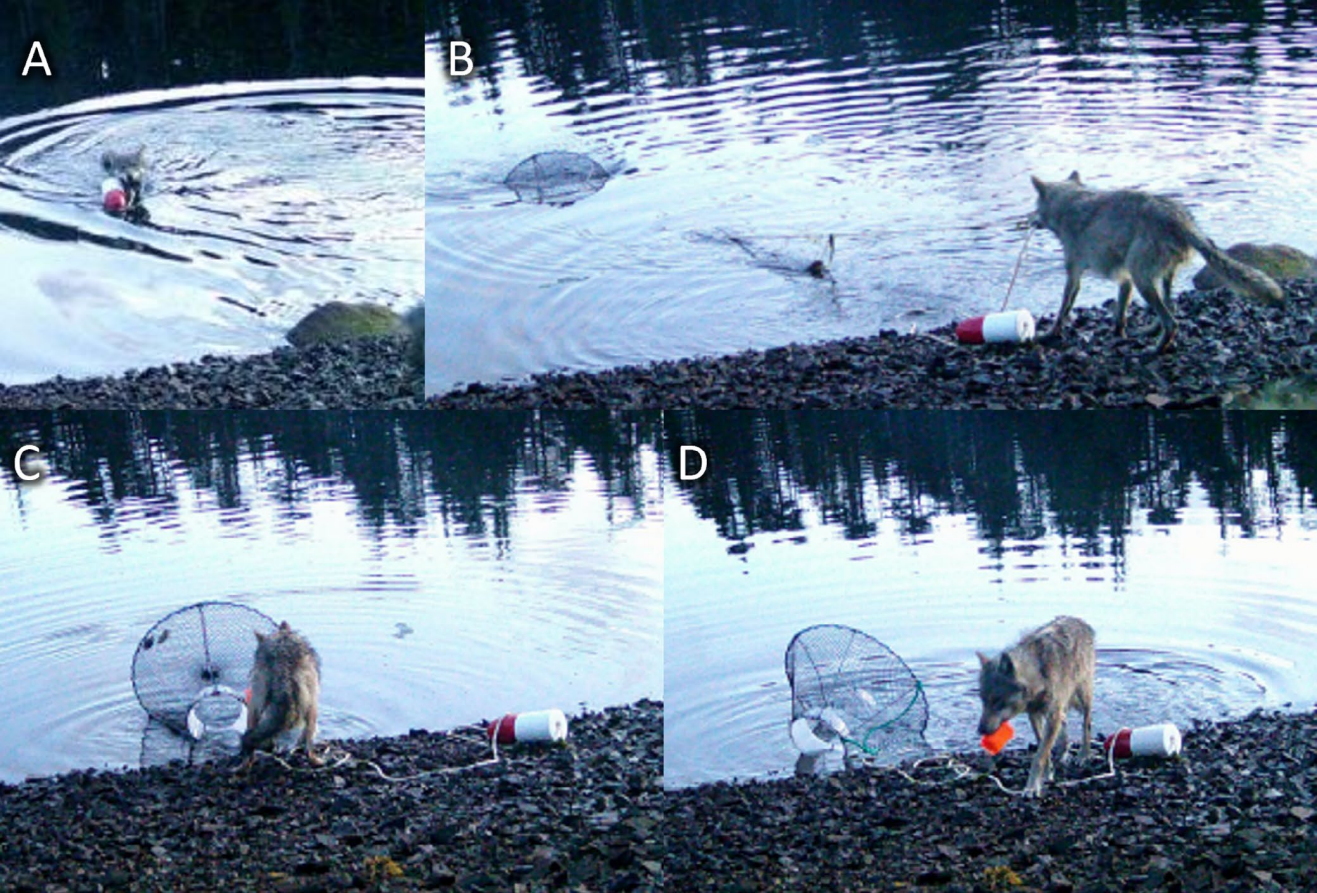
(A–D) Stills extracted from remote camera video of a wolf in Haíɫzaqv Territory pulling an initially submerged green crab trap to shore to access baited cup within. Observation recorded on May 29, 2024 (not April –erroneous date set on camera). See Video 1 for full recording.

**VIDEO 1:** Remote camera video (five separate sequential videos combined) of a wolf in Haíɫzaqv Territory pulling an initially submerged green crab trap to shore to access baited cup within. Observation recorded on May 29, 2024 (not April—erroneous date set on camera). Video content can be viewed at https://onlinelibrary.wiley.com/doi/10.1002/ece3.72348.

This sequence appears to demonstrate a sophisticated understanding of the multi‐step connection between the floating buoy and the bait within the out‐of‐sight trap. Alternative explanations for this behavior could be proposed that do not involve causal insight: for example, complex behaviors can often be explained by simpler processes such as trial‐and‐error learning (Shettleworth 2010), as when insects learn to use tool‐like behaviors to access unseen foods (Giurfa 2013; Alem et al. 2016). However, when nonhuman animals exhibit multi‐step behaviors repeatedly and efficiently, the most parsimonious explanation might include at least some causal understanding (Shettleworth 2010) as would be assumed for a human in similar circumstances (de Waal 1999). In this case, the trap was fully submerged and not visible from shore, suggesting that the wolf recognized that the buoy was attached to a rope, in turn attached to an unseen trap containing edible bait. She appeared to understand that these components could be pulled in sequence to progressively retrieve the trap from the water and obtain the bait that was presumably within. The focus exhibited during this encounter appeared ‘unwaveringly purposeful’, the term used by Köhler (1917) for describing the seemingly intentional actions employed by chimpanzees when solving novel problems. The efficiency and speed of the sequence, combined with observations of other similarly moved and damaged traps in the area, suggest previous experience and intent. Regardless of the extent to which the wolf truly understood the trap's mechanics, whether this behavior reflected sophisticated understanding or more simplistic trial‐and‐error learning, the very act of appropriating human tools to achieve a goal is noteworthy. By analogy, these very words were typed on a computer whose inner workings the authors do not fully understand, yet we believe (and hope) our use of them suggests that we too possess some measure of higher cognition.

Whether the trap‐pulling behavior observed here qualifies strictly as tool use might vary depending on the definition. Tool use is typically understood as using an external object to achieve a specific goal with intent (Bentley‐Condit and Smith 2010; Shumaker et al. 2024) – a definition argued to include even stick chewing by dogs (Brooks and Yamamoto 2021). However, other definitions exclude rope pulling as a form of tool use, “because they are not responsible to the proper and effective orientation of the tool to the incentive”, and that for this to qualify as tool use “the animal must produce, not simply recognize, the proper and effective orientation between the tool and the incentive” (Shumaker et al. 2024). The sophistication of this particular sequence might suggest an exception to the rope pulling exclusion—as might the fact that pulling the rope is the key mechanism for retrieving crab traps even by humans.

This observation raises questions about the origins and context of this behavior. We currently lack evidence to determine whether this is the only wolf exhibiting this behavior, or if it has been shared among other wolves. We have installed permanent remote camera stations in the area, but they have not yet captured an additional fully submerged trap being pulled from the water.

On February 14, 2025, a different individual was recorded pulling a line attached to a partially submerged trap. The camera was triggered 8 min later when that and an additional originally out‐of‐frame trap of unknown initial depth were on the beach with bait cups removed (Video 2). However, we do not know if this individual has learned to extract fully submerged traps.

**VIDEO 2:** Remote camera video (three separate sequential videos combined, with delay of approximately 8 min between camera being triggered between first and second videos, and 2 min between second and third) of a wolf in Haíɫzaqv Territory pulling partially submerged green crab trap to shore to access baited cup within. Observation recorded on February 14 2025. Video content can be viewed at https://onlinelibrary.wiley.com/doi/10.1002/ece3.72348.

Other traps have intermittently been damaged and dragged nearby, though not recorded on camera. The origin of this behavior remains uncertain. It is possible that she or another wolf learned by observing Haíɫzaqv Guardians pulling traps, though Guardians lift traps vertically out of the water from boats, not horizontally to shore. Alternatively, many traps are exposed and easily accessible at low tides. This behavior might have been learned incrementally, initially targeting fully exposed traps, then targeting slightly submerged traps (as in the 2025 observation), and ultimately retrieving fully submerged traps, including those in water too deep to ever be exposed.

More broadly, we cannot ascertain whether this level of sophisticated behavior is more common than previously assumed but rarely documented due to the elusive and rarely observed nature of noncaptive wolves in general, and of family units in this area more specifically, consistent with captivity bias predictions (Haslam 2013). We similarly do not know if the preconditions for such behavior are universal among noncaptive wolves or more specific to wolves in this region. For example, wolves in this area face relatively low levels of human persecution (e.g., hunting and trapping), which is rare globally (Tallian et al. 2023; Morales‐González et al. 2025). Reduced need for vigilance might allow wolves to develop confidence and devote time to exploring novel behaviors such as those observed in this study, which might be less expected in more persecuted populations prioritizing vigilance. This explanation would be consistent with canids elsewhere: for example, wolves often limit their activities to avoid humans spatially or temporally (Wam et al. 2012; Martínez‐Abraín et al. 2023; Smith et al. 2024), while coyotes exhibit more exploratory behavior in urban environments where persecution is rare compared to rural environments where it is more common (by humans or other predators; Breck et al. 2019).

The cognitive sophistication seemingly exhibited here might prompt further ethical considerations. In many species, perception of sophisticated intelligence is positively associated with the assumed duty of care and consideration (Piazza and Loughnan 2016). While acknowledging cautions regarding overinterpretation of tool use as indicators of intelligence (Emery and Clayton 2009; Shumaker et al. 2024), the sophisticated intelligence suggested here might evoke reconsideration of common negative perceptions toward this species (perceptions that are not unanimous and not representative of the Haíɫzaqv Nation—see https://www.kindredpodcast.co/48‐coastal‐sea‐wolves‐of‐bella‐bella‐a‐conversation‐with‐william‐housty‐of‐the‐heiltsuk‐nation/), including elsewhere in British Columbia where wolves are still killed by the provincial government (Darimont and Paquet 2024). Moreover, if the capacity to develop this behavior was potentially facilitated by relatively low levels of human persecution, it raises additional questions about the consequences of such persecution, which likely extend beyond the primarily demographic focus of most wildlife management (Ordiz et al. 2013; Bryan et al. 2014; Cassidy et al. 2023).

Notably, observing this behavior was only possible due to the Haíɫzaqv Guardian Program, the only entity that conducts research and monitoring here year‐round (Artelle et al. 2022). Following the “M̓ṇúxvʔit model” (White (Q̓íx̌itasu) et al. 2024)—which centers Indigenous Knowledge and Governance in collaborations—this work was invited by HIRMD, who asked the question ‘who is attacking the traps?’ This observation exemplifies the kinds of insights into the ecology of this region, and into species biology more broadly, that are enabled by the robust monitoring and research work led by, and/or in collaboration with, Indigenous Guardians, and by the Haíɫzaqv governance guiding informed work in this territory. The Haíɫzaqv Wolf and Biodiversity Project research program—a collaborative effort between SUNY ESF and the Heiltsuk Integrated Resource Management Department, and supported by the Woodland Park Zoo and others—aims to further elucidate the ecology, dynamics, and biocultural context of wolves here, including some of the questions raised herein.

## Author Contributions

**Kyle A. Artelle:** conceptualization (lead), data curation (lead), project administration (lead), visualization (lead), writing – original draft (equal), writing – review and editing (equal). **Paul C. Paquet:** writing – original draft (equal), writing – review and editing (equal).

## Conflicts of Interest

The authors declare no conflicts of interest.

## Data Availability

All reported data (recorded observations) are provided within the manuscript. The exact location of the observations are sensitive and cannot be provided publicly in accordance with data sharing agreements with the Haíɫzaqv Nation and to protect the species.
